# 804 Catastrophic Burn Management: A case series using autologous skin cell suspension

**DOI:** 10.1093/jbcr/irac012.353

**Published:** 2022-03-23

**Authors:** Lyndsay Deeter

**Affiliations:** Western States Burn Center, North Colorado Medical Center, Greeley, Colorado

## Abstract

**Introduction:**

Catastrophic thermal injury treatment is complex due to the lack of autologous donor site, which is imperative for permanent wound closure. Historically, our burn unit has relied on application of cultured epithelial autografts for wound closure in this patient population. Lab grown skin requires a significant time investment. Therefore, in our most recent subset of large burn injuries, we have transitioned to the use of autologous skin cell suspension over widely meshed autograft utilization almost exclusively.

**Methods:**

A case series of four thermally injured patients with injuries greater than 75% TBSA was reviewed. Patient length of stay, operative interventions, skin coverage, mortality and cost were examined.

**Results:**

Burn size ranged from 75-85% TBSA in four patients. Early excision was implemented for all four patients in the case study. One of the four patients was treated with a combination of lab grown skin and autologous skin cell suspension. The three other patients were treated exclusively with autologous skin cell suspension for wound coverage. All patients underwent widely meshed autograft with the autologous cell suspension for wound coverage. All four patients survived to discharge and their initial follow up. Cost of operative interventions were less expensive for autologous skin cell suspension than for cultured epithelial autografts. Length of stay varied from 66-179 days. Discharge location varied from acute rehabilitation, to a homeless shelter, to another burn facility with inpatient psychiatric services.

**Conclusions:**

Autologous skin cell suspension is a safe and efficient treatment modality to obtain permanent wound closure in catastrophic thermal injuries.

